# The curse of experiencing and committing violence as a criminal recidivism predictor: A comparison between female forensic psychiatric patients with severe mental disorders and substance use disorder

**DOI:** 10.1192/j.eurpsy.2023.2450

**Published:** 2023-09-04

**Authors:** Michael Fritz, Georgios Karanassios, Viviane Wolf, Juliane Mayer, Ivonne Steiner, Irina Franke, Verena Klein, Judith Streb, Manuela Dudeck

**Affiliations:** 1 Department of Forensic Psychiatry and Psychotherapy, Ulm University, Ulm, Germany; 2 School of Health and Social Sciences, AKAD University of Applied Sciences, Stuttgart, Germany; 3 Department of Psychiatry and Psychotherapy, Medical Faculty, LVR-Clinic Duesseldorf, Duesseldorf, Germany; 4 Department of Forensic Psychiatry and Psychotherapy, kbo-Isar-Amper-Clinic Taufkirchen (Vils), Taufkirchen (Vils), Germany; 5 Forensic Psychiatry, Psychiatric Services of Grisons, Chur, Switzerland

**Keywords:** drug addiction, female aggression, forensic psychiatry, severe mental disorders, violent experiences

## Abstract

**Background:**

Violence occurs frequently in the life of forensic psychiatric patients, both as active aggression and in the form of victimization. Undoubtedly, these incidents shape personality, behavior, and affect the ability to interact adequately socially. Thus, such experiences may influence criminal recidivism and serve as forensic psychiatric/psychological predictors upon hospital discharge.

**Methods:**

Hence, this study aimed at characterizing two distinct female forensic psychiatric patient populations (nonsubstance use mental disorders [*n* = 110] versus substance use disorder [*n* = 415]) regarding their active and passive violent experiences as well as contextualizing these with their individual crime recidivism rates. The analysis followed a record-based, retrospective approach.

**Results:**

While both groups experienced aggression throughout childhood and youth equally often, substance use disorder patients were significantly more often exposed to violence during adulthood. On the other hand, severely mentally ill patients tended to react more often with violence during their hospital confinement. However, regarding their violent recidivism rate, no intergroup effects were observed. Finally, within the addicted group, a violent index crime as well as physical aggression during hospital confinement increased the odds for violent reoffending by approximately 2.4-fold (95% confidence interval 1.3–4.5) and 2.5-fold (95% confidence interval 1.1–5.9), respectively.

**Conclusion:**

In summary, these findings underline the importance of active aggression rather than victimization as an influencing factor on resocialization especially in a substance use disorder patient population.

## Introduction

In Germany, involuntary commitment to a forensic psychiatric institution requires conviction under Section 20 (not guilty for reasons of insanity; https://www.gesetze-im-internet.de/englisch_stgb/englisch_stgb.html#p0153) or Section 21 (guilty, but with severely diminished criminal responsibility; https://www.gesetze-im-internet.de/englisch_stgb/englisch_stgb.html#p0155) of the German Criminal Code. Additionally, a hospital treatment order (Section 63, German Criminal Code; https://www.gesetze-im-internet.de/englisch_stgb/englisch_stgb.html#p0433) must come into effect if it is likely that additional severe crimes related to severe mental disorder may be committed by the same person in the future [1, 2]. Alternatively, in offenders with substance use disorder (SUD), there must be a link between crime and habitual drug consumption combined with reasonable belief in therapeutic success to prevent further crimes (Section 64, German Criminal Code; https://www.gesetze-im-internet.de/englisch_stgb/englisch_stgb.html#p0435 [2]). For a detailed description of the legal framework, the authors recommend to read the work of Müller-Isberner et al. [1]. Consequently, German forensic psychiatric patient populations comprise of both female and male individuals with a diagnosis of either a mental illness predominantly of schizophrenia spectrum disorders (SSDs; 41%), SUDs, or a combination of both [1, 3]. Both SSDs as well as SUDs are linked to heightened criminally conspicuous behavior such as violence and aggression [4–6]. Thus, around 60% of Section 63 patients as well as ~62% (alcohol) and ~ 14% (other substances) of Section 64 patients have a conviction due to violent crimes [7, 8]. Patients with SSD in particular account for the highest rates of violence among all mentally ill individuals [4, 9], while additional substance abuse further increases the risk for violence and reoffending [10, 11].

From a bio-psycho-social model’s perspective, one of the main factors promoting violence in both sexes/genders alongside biological modulators [12] and traits [13, 14] are adverse childhood experiences (ACEs; [15]). ACEs represent different forms of abuse (psychological, physiological, or sexual) or neglect (e.g., a dysfunctional household) experienced during a child’s upbringing. While gender differences in the impact of ACEs on violent behavior remain unclear, ACEs are overall linked to an increased probability of developing SUDs [16] or SSDs [17], as well as an increased risk of becoming violent offenders [18]. Leban and Delacruz [18] specifically noted increased odds of violent delinquency in both boys and girls having experienced ACEs but also stated this effect to be more pronounced in boys. However, Been et al. [19] referred to it as an archaic belief that women would not readily engage in violence, even though it is noteworthy that there is some evidence men and women express aggression differently [20].

Hence, it is not surprising that active (violent offenses, inpatient violence) and passive experiences of violence during adult- or childhood appear to be common among the two patient populations. With the main treatment goal being the prevention of new crimes, understanding the underlying psychological factors may be crucial for effective treatment and management. However, despite significant progress in the field, there is a lack of research comparing the experiences of violence between female forensic psychiatric patients with severe mental illness and those with acute SUD.

To address this gap, the present study aims to compare the active and passive lifetime experiences of physical violence/abuse in female forensic psychiatric patients with mental illness and those with acute SUD. The present study is also aimed at examining these patients’ lifetime experiences with violence trying to identify risk and prognostic factors for renewed violent crimes in each group.

## Methods

### Patient sample

The sample included all female patients who were legally admitted to the forensic psychiatric hospital in Taufkirchen (Germany) and were discharged between January 1, 2001, and December 31, 2017. In Germany, admission to a forensic psychiatric hospital is based on a court decision in accordance with Section 63 or 64 of the German Criminal Code. If a person commits a serious criminal offense due to a mental disorder and there is a high risk of recidivism, the court orders that person’s placement pursuant to Section 63 of the German Criminal Code. The length of hospitalization is not limited by law. Placement under Section 64 of the Criminal Code requires a diagnosis of SUD, a high risk of new crimes to be committed, and a favorable treatment prognosis; it has a standard duration of 2 years. In total, the data records of 557 women were collected. In total, 32 incomplete records were excluded from further analysis, resulting in 525 patients analyzed. A total of 415 patients were hospitalized according to Section 64 of the German Criminal Code and 110 patients according to Section 63.

Recidivism occurrence rates were determined through the German Criminal Federal Central Register records. Each entry was considered a recidivism if the date of the offense followed the hospital discharge date, and an assessment was also made to determine if it constituted a violent reoffence. The HCR-20 v3 manual [21] was used to define an act of violence. It is comprised of three scales from which it derives its acronym; the Historical scale, the Clinical scale, and the Risk Management scale. The Historical scale gathers information about previous violence, the Clinical scale collects information of clinical relevance regarding the mental state of the subject, and the Risk Management scale integrates this information into a prognostic assessment for future risks regarding violence. In comparison to the Violence Risk Appraisal Guide-Revised (VRAG-R) [22], another commonly used instrument for the prognosis of violent behavior, the HCR-20 v3 has a broader definition of “violent recidivism” and includes, for example, the mere threat of violence. The observation period for reoffending from the time of release until the first recidivism or the end date of the survey (if there was no new crime committed) was on average 6 years (*SD* = 4.90). Further descriptive data can be found in Table 1. An ethics vote for this study was obtained from the Bavarian Medical Association (No. 2019-167).

**Table 1.** Sample description

	Non-SUD mental disorders (*n* = 110) *M* (*SD*)/*n* (%)	Substance use disorder (*n* = 415) *M* (*SD*)/*n* (%)	Statistics
Age at admission (years)	39.48 (12.12)	32.73 (9.05)	*t*(523) = 6.44, *p* < .001
Age at first manifestation (years)	26.26 (12.90)	18.14 (7.18)	*t*(523) = 8.72, *p* < .001
Age at first conviction (years)	36.32 (13.25)	25.45 (9.20)	*t*(522) = 8.10, *p* < .001
Number of previous inpatient treatments	7.67 (15.00)	5.13 (7.22)	*Z* = −1.291, *p =* .197
Duration of admission	63.14 (27.88)	23.77 (13.81)	*Z* = −13.440, *p* < .001
*Diagnosis*			Chi^2^(12) = 409.742, *p* < .001
Organic disorders (F06.–07.)	5 (5%)	3 (1%)	
Schizophrenia or schizotypal disorders (F20.–29.)	69 (63%)	1 (0.2%)	
Mood disorders (F30.–39.)	9 (8%)	2 (0.5%)	
Personality disorders (F60.–F61.)	18 (16%)	29 (7%)	
Neurotic or stress-related disorders (F40.–43.)	2 (2%)	0	
Mental retardation (F70.–79.)	4 (4%)	1 (0.2%)	
*Mental and behavioral disorders due to the use of*			
…alcohol (F10.)	1 (1%)	70 (17%)	
…opioids (F11.)	0	79 (19%)	
…cannabinoids (F12.)	1 (1%)	9 (2%)	
…sedatives or hypnotics (F13.)	1 (1%)	4 (1%)	
…cocaine (F14.)	0	5 (1%)	
…other stimulants (F15.)	0	44 (11%)	
…multiple drug use (F19.)	0	168 (41%)	
*Highest school degree*			Chi^2^(3) = 24.970, *p* < .001
No educational qualification	28 (26%)	82 (20%)	
*Completed school to the end*			
…of grade 9	36 (33%)	235 (57%)	
…of grade 10	25 (23%)	71 (17%)	
Graduated high school	19 (18%)	26 (6%)	
Time at risk (months)	102.10 (54.17)	64.13 (57.39)	*t*(523) = 6.24, *p* < .001

The present study was part of a larger project regarding the applicability of a common risk assessment instrument in female forensic inpatients. The codebook (i.e., assessment instrument) presently used was designed in collaboration with the Office of Corrections and Rehabilitation, Zurich, Switzerland. It provided item definitions as well as a respective rating scheme, serving as a detailed coding guide for the included items (i.e., sociodemographic data, gender-responsive risk factors, and risk assessment instruments). Following a detailed literature review, gender-specific risk factors with sufficient empirical evidence were selected. These factors included the following variable domains: mental health (e.g., diagnoses); trauma/victimization (e.g., experiences of sexual violence during childhood); intimate partner dysfunction (e.g., unstable intimate relationship); parental stress (e.g., loss of child custody); self-esteem (low self-esteem); and poverty (e.g., homelessness). Diagnoses were coded according to the ICD-10 criteria. The item definitions were mainly created based on the available literature.

### Statistics

Statistical analyses were performed using IBM SPSS Statistics Version 28. First, comparisons between patients with severe mental disorders and SUD were conducted for all outcomes (i.e., index offense, diagnosis of dissocial personality disorder, physical violence experiences in adulthood and in childhood/adolescence, inpatient violence, and violent recidivism) using separate chi-square tests of independence. Cramer’s V was used as a measure of effect size. Following Cohen’s [23] guidelines for interpretation, effect sizes below .10 were regarded as small, those between .10 and .30 as medium, and those above .30 were considered large. Differences in the time to reoffending between patients with severe mental disorders and SUD were analyzed with the Kaplan–Meier estimator. Two separate binary logistic regression analyses were performed to determine the specific contribution of the examined factors in predicting a violent offense. The first analysis focused on female patients with severe mental illness, while the second analysis considered patients with SUDs.

## Results

A first analysis approach revealed patients with non-SUD mental disorders to be significantly more likely to commit a violent crime (i.e., homicide, assault, robbery, or arson; 84%, 92 of 110) than addicted individuals (32%, 133 of 415, Chi^2^(5) = 130.825, *p* < .001, Cramer-*V* = .499, see Table 2).

**Table 2.** Index offense in the two groups

	Non-SUD mental disorders (*n* = 110)	Substance use disorder (*n* = 415)	Total (*N* = 525)
Index offense			
Homicide	25 (23%)	21 (5%)	46
Assault	37 (34%)	75 (18%)	112
Robbery	11 (10%)	27 (7%)	38
Arson	19 (17%)	10 (2%)	29
Drug	0	201 (48%)	201
Other	18 (16%)	81 (20%)	99

Analysis of the frequency of an antisocial/dissocial personality disorder diagnosis (ICD-10: F60.2) shows that both groups displayed equally high occurrence rates (non-SUD mental disorder patients 4%, 4 of 110; addicted patients 7%, 27 of 415 [Chi^2^(1) = 1.289, *p* = .363, Cramer-*V* = .050]).

However, in their adult life, significantly more patients with SUD reported experiencing physical violence (see Table 3, Chi^2^(1) = 13.369, *p* < .001, Cramer-*V* = .161). On the other hand, analysis of physical violence experience during childhood and adolescence shows no variations between both patient groups (see Table 3; Chi^2^(1) = .282, *p =* .595, Cramer-*V* = −.024).

**Table 3.** Physical violence experiences in child- and adulthood in the two groups

	Non-SUD mental disorders (*n* = 110)	Substance use disorder (*n* = 415)	Total (*N* = 525)
*Physical violence experiences (by family members, partners, friends, or strangers)*			
In adulthood	38 (36%)	227 (56%)	265 (52%)
In childhood/adolescence	55 (55%)	206 (52%)	261 (52%)

Furthermore, patients with non-SUD mental disorders were significantly more likely to use physical violence against objects, fellow patients, or staff during their hospital placement than patients with SUD (Chi^2^(3) = 48.891, *p* < .001, Cramer-*V* = −.276, see Table 4).

**Table 4.** Physical violence during detention in the two groups

	Non-SUD mental disorders (*n* = 110)	Substance use disorder (*n* = 415)	Total (*N* = 525)
*Physical violence during detention*			
No	73 (66%)	375 (90%)	448 (85%)
Against objects	7 (6%)	13 (3%)	20 (4%)
Against fellow patients and staff	12 (11%)	18 (4%)	30 (6%)
Against objects, fellow patients, and staff	18 (16%)	9 (2%)	27 (5%)

Somewhat surprisingly, the two patient populations did not differ regarding their relapse rates for violent crimes (that is violent recidivism) after discharge (see Table 5, Chi^2^(1) = 1.449, *p* = .245, Cramer-*V* = .053), even after taking into account the different lengths of time at risk (Table 6).

**Table 5.** Recidivism with a violent crime in the two groups

	Non-SUD mental disorders (*n* = 110)	Substance use disorder (*n* = 415)	Total (*N* = 525)
*Recidivism with a violent crime*			
No	101 (92%)	364 (88%)	465 (89%)
Yes	9 (8%)	51 (12%)	60 (11%)

**Table 6.** Results of the binary logistic regression predicting recidivism with a violent offense for patients with SUDs compared to patients with mental disorders other than SUDs taking into account the time at risk

	Odds-ratio	95%-confidence interval
Time at risk	.750*	.669–.840
SUD (=1) versus non-SUD (=0)	.674	.295–1.541

* *p* < .05.

Finally, the results of the binary logistic regression for predicting recidivism with a violent offense (1 = yes, 0 = no) can be found in Table 7. For female offenders with non-SUD mental disorders, violent recidivism cannot be predicted based on the studied factors: a violent index offense, diagnosis of dissocial personality disorder, physical violence experiences in adulthood or childhood/adolescence, and physical violence during detention (1 = yes, 0 = no). However, addicted female offenders were 2.4 times more likely to engage in violent reoffending when they already committed a violent offense as an index crime, and 2.5 times more likely when they have exhibited violence against fellow patients, staff, or objects during treatment.

**Table 7.** Results of the binary logistic regression predicting recidivism with a violent offense for patients with SUDs compared to patients with mental disorders other than SUDs

	Odds-ratio	95%-confidence interval
*Severe mental disorder*		
Violent offense as index offense	1.468	.277–7.780
Dissocial personality disorder	.011	.001–.012
*Physical violence experiences*		
…in adulthood	2.357	.554–10.022
…in childhood/adolescence	.995	.233–4.244
Physical violence during detention	.247	.029–2.097
*Substance use disorder*		
Violent offense as index offense	**2.375** *	**1.250–4.513**
Dissocial personality disorder	1.319	.449–3.878
*Physical violence experiences*		
…in adulthood	1.203	.613–2.361
…in childhood/adolescence	1.170	.602–2.275
Physical violence during detention	**2.540** *	**1.093–5.903**

* *p* < .05.

The exact p values are .008 and .030.

## Discussion

The present findings demonstrated that the female SUD patient population in a German forensic psychiatric clinical setting had experienced more violence during adulthood than patients with other mental illnesses than SUD. It is, however, noteworthy that no differences at earlier life stages were found. Even though patients with non-SUD mental disorders were more likely to have a violent index crime and to use violence against objects, fellow patients, and staff, their violent recidivism rate did not differ from the drug addiction patient group. On the contrary, SUD patients increased their odds for a violent reoffending by approximately 2.5-fold when the index crime was violent or physical aggression occurred during the involuntary confined treatment.

Clinical data suggests that female SUD forensic psychiatric patients experience certain ACEs (sexual, verbal, and emotional abuse and emotional neglect) more often than men [24, 25]. On the other hand, there is some evidence that ACEs affect men more profoundly than women [18], although Pflugradt et al. recently demonstrated an increased incidence of ACEs in female murderers [26]. Knowing that ACEs increase the probability of becoming violent offenders [18] and developing other health issues [16, 17], this may still explain why violent index crimes are more prominent in male forensic psychiatric patients [24]. However, our current findings suggest that physical abuse during childhood and youth adds very little to no prognostic value regarding violent reoffending or the duration until criminal recidivism in both female patient populations. Surprisingly, similar findings were observed when looking at violence during their hospital confinement and violence as an index crime in patients with other mental illnesses than SUD, thereby contradicting findings on male patients [27].

Also, a codiagnosis of antisocial/dissocial personality disorder in either group did not impact the time interval to a first reoffence or overall violent recidivism rates, further indicating possible gender differences regarding personality disorders and violent crimes [28].

While other studies identified alcohol as one of the main factors influencing reoffending and violent crimes overall in forensic psychiatric patient populations in both sexes/genders [29–31], we were able to extend these prognostic factors. Particularly, the present findings show that a violent index crime as well as physical aggression during the hospitalization period did indeed serve as negative predictors of violent reoffending in the addiction group. However, these parameters had no influence on violent recidivism in patients with non-SUD mental disorders.

The surprising observation that both patient populations did not differ regarding their relapse rates for violent crimes (8% §63 vs. 12% §64), however, may not be that astonishing at all and rather be based on a subconscious psychological perception bias. A bias, in fact, the authors fell victim to as well. One may simply believe that §63 patients are more dangerous. Our current data though aligns well with the observations made by Harrerdorf, even across sexes/genders [32]. In this study, a similar violent crime recidivism rate in two male forensic psychiatric patient populations was reported (8.5% [§63] vs. 19.2% [§64]).

A likely hypothesis that could explain this discrepancy may be the different nature of aggression in both groups. For instance, in patients with SSDs, aggression is augmented by delusions [33] and could hence be seen as reactive [34] to an imaginary threat. Among SUD patients showing aggression, it may very well be a learning effect of utilizing violence to reach certain aims (i.e., instrumental aggression; [35]). In such a case, aggression would readily depend on medication compliance in one group but be a strategic mean of reaching goals in the other, thus explaining the different predictive values. However, further comparative research is needed to prove such a hypothesis since other risk factors may contribute as well. Such risk factors may be personality disorders [36] including the impulsive and borderline type [37, 38], as well as a poor self-image [39] and low self-efficacy [40].

Nonetheless, each scientific work has its limitations and so does this one. The primary limitation is that the present study is based on a retrospective analysis of patient records only. Hence, information was exclusively retrieved from pre-existing patient records, limiting any confirmation of accuracy in content and completeness due to differences in the records used.

Another limitation is the exclusive reliance on external assessments. Patients’ perspectives were not included in the present study, as there was no direct patient–researcher interaction.

Despite such limitations, the study draws on a major strength, namely the fact that the study sample represents a complete survey of female patients treated in a forensic psychiatric facility in Bavaria over the period of 17 years. This makes the present work one of the largest investigations in female forensic patients existing, since so far comparable studies reported significantly smaller numbers of participants [25, 41, 42].

## Data Availability

The raw data supporting the conclusions of this manuscript will be made available by the authors, without undue reservation, to any qualified researcher. Requests to access the datasets should be directed to judith.streb@uni-ulm.de.
